# Association of TNFAIP3 interacting protein 1, TNIP1 with systemic lupus erythematosus in a Japanese population: a case-control association study

**DOI:** 10.1186/ar3134

**Published:** 2010-09-17

**Authors:** Aya Kawasaki, Satoshi Ito, Hiroshi Furukawa, Taichi Hayashi, Daisuke Goto, Isao Matsumoto, Makio Kusaoi, Jun Ohashi, Robert R Graham, Kunio Matsuta, Timothy W Behrens, Shigeto Tohma, Yoshinari Takasaki, Hiroshi Hashimoto, Takayuki Sumida, Naoyuki Tsuchiya

**Affiliations:** 1 Molecular and Genetic Epidemiology Laboratory, Doctoral Program in Life System Medical Sciences, Graduate School of Comprehensive Human Sciences, University of Tsukuba, 1-1-1 Tennodai, Tsukuba, Ibaraki 305-8575, Japan; 2 Division of Clinical Immunology, Doctoral Program in Clinical Sciences, Graduate School of Comprehensive Human Sciences, University of Tsukuba, 1-1-1 Tennodai, Tsukuba, Ibaraki 305-8575, Japan; 3 Department of Rheumatology, Niigata Rheumatic Center, 1-2-8 Hon-cho, Shibata, Niigata, 957-0054 Japan; 4 Department of Rheumatology, Clinical Research Center for Allergy and Rheumatology, Sagamihara National Hospital, National Hospital Organization, 18-1 Sakuradai, Minami-ku, Sagamihara, Kanagawa 252-0392, Japan; 5 Division of Rheumatology, Department of Internal Medicine, Juntendo University, 2-1-1 Hongo, Bunkyo-ku, Tokyo 113-8421, Japan; 6 Immunology Biomarkers Group, Genentech, Inc. 1 DNA Way, South San Francisco, California 94080-4990, USA; 7 Matsuta Clinic, 2-28-8 Daizawa, Setagaya-ku, Tokyo 115-0032, Japan; 8 Juntendo University School of Medicine, 2-1-1 Hongo, Bunkyo-ku, Tokyo 113-8421, Japan

## Abstract

**Introduction:**

*TNFAIP3 *interacting protein 1, *TNIP1 *(ABIN-1) is involved in inhibition of nuclear factor-κB (NF-κB) activation by interacting with TNF alpha-induced protein 3, A20 (*TNFAIP3)*, an established susceptibility gene to systemic lupus erythematosus (SLE) and rheumatoid arthritis (RA). Recent genome-wide association studies revealed association of *TNIP1 *with SLE in the Caucasian and Chinese populations. In this study, we investigated whether the association of *TNIP1 *with SLE was replicated in a Japanese population. In addition, association of *TNIP1 *with RA was also examined.

**Methods:**

A case-control association study was conducted on the *TNIP1 *single nucleotide polymorphism (SNP) rs7708392 in 364 Japanese SLE patients, 553 RA patients and 513 healthy controls.

**Results:**

Association of *TNIP1 *rs7708392C was replicated in Japanese SLE (allele frequency in SLE: 76.5%, control: 69.9%, *P *= 0.0022, odds ratio [OR] 1.40, 95% confidence interval [CI] 1.13-1.74). Notably, the risk allele frequency in the healthy controls was considerably greater in Japanese (69.9%) than in Caucasians (24.3%). A tendency of stronger association was observed in the SLE patients with renal disorder (*P *= 0.00065, OR 1.60 [95%CI 1.22-2.10]) than in all SLE patients (*P *= 0.0022, OR 1.40 [95%CI 1.13-1.74]). Significant association with RA was not observed, regardless of the carriage of human leukocyte antigen DR β1 (*HLA-DRB1*) shared epitope. Significant gene-gene interaction between *TNIP1 *and *TNFAIP3 *was detected neither in SLE nor RA.

**Conclusions:**

Association of *TNIP1 *with SLE was confirmed in a Japanese population. *TNIP1 *is a shared SLE susceptibility gene in the Caucasian and Asian populations, but the genetic contribution appeared to be greater in the Japanese and Chinese populations because of the higher risk allele frequency. Taken together with the association of *TNFAIP3*, these observations underscore the crucial role of NF-κB regulation in the pathogenesis of SLE.

## Introduction

*TNFAIP3 *(tumor necrosis factor α-induced protein 3) encodes a ubiquitin-editing protein, A20, known as an inhibitor of nuclear factor-κB (NF-κB). Several adaptor molecules are thought to associate with A20 and be involved in inhibition of NF-κB [1]. TNIP1 (TNFAIP3 interacting protein 1), also known as ABIN (A20-binding inhibitor of NF-κB)-1, is one such adaptor molecule binding to A20. *TNIP1 *mRNA is strongly expressed in peripheral blood lymphocytes, spleen and skeletal muscle, and the expression is also detected in kidney [2]. *TNIP1 *expression is induced by NF-κB, and in turn, overexpression of *TNIP1 *inhibits NF-κB activation by TNF [1], although deficiency of *TNIP1 *has few effects on NF-κB inhibition [3]. Thus, TNIP1 appears to play a role in NF-κB inhibition, at least partly by interacting with A20. In addition, TNIP1 was shown to inhibit TNF-induced apoptosis independently of A20 [3].

*TNFAIP3*, located at 6q23, has been identified as a susceptibility gene for both systemic lupus erythematosus (SLE) and rheumatoid arthritis (RA) in Caucasian and Asian populations [4-8]. Recently, Shimane et al. [9] replicated association of *TNFAIP3 *single nucleotide polymorphisms (SNPs) with SLE and RA in a Japanese population. We also detected association of *TNFAIP3 *rs2230926 with Japanese SLE patients in an independent study [10].

Recently a genome-wide association study (GWAS) reported association of *TNIP1 *(5q32-q33.1) as well as *TNFAIP3 *SNPs with psoriasis in the Caucasian populations [11]. Subsequently, two recent GWAS revealed association of *TNIP1 *intronic SNPs rs7708392 and rs10036748, which are in strong linkage disequilibrium (LD) with SLE in the Caucasian (European-American and Swedish) and Chinese Han populations, respectively [8,12]. These observations underscored the role of the pathway involving *TNFAIP3-TNIP1 *in the genetic predisposition to SLE. The association of *TNIP1 *with SLE needs to be further confirmed.

Recently, it has become increasingly clear that SLE and RA share a number of susceptibility genes. *TNFAIP3 *[4-10], *STAT4 *[13,14] and *BLK *[15,16] represent such shared susceptibility genes. *TNIP1 *has been shown to be upregulated in synovial tissues from RA [17], raising a possibility that *TNIP1 *may also play a role in the pathogenesis of RA. To date, association of RA with *TNIP1 *has not been reported.

This study was conducted to examine whether *TNIP1 *was associated with SLE and RA in a Japanese population.

## Materials and methods

### Patients and controls

Three hundred sixty-four Japanese patients with SLE (21 males and 343 females, mean ± SD age, 42.8 ± 13.9 years), 553 patients with RA (43 males and 510 females, mean ± SD age, 58.0 ± 11.3 years) and 513 healthy controls (238 males and 275 females, mean ± SD age, 34.1 ± 9.9 years) were recruited at University of Tsukuba, Juntendo University, Sagamihara National Hospital, Matsuta Clinic and the University of Tokyo (Table 1). All patients and healthy individuals were native Japanese living in the central part of Japan. All patients with SLE and RA fulfilled the American College of Rheumatology criteria for SLE [18] and RA [19], respectively. Consecutive patients ascertained in rheumatology specialty hospitals or clinics were recruited. The patients with SLE were classified into subgroups according to the presence or absence of renal disorder, neurologic disease and serositis based on the definition of ACR criteria [18], anti-dsDNA and anti-Sm antibodies, and age of onset (< 20 yr). The numbers of the missing data were 5 (renal disorder), 3 (neurologic disease), 21 (serositis), 19 (anti-dsDNA antibody), 22 (anti-Sm antibody) and 6 (age of onset). Patients with RA and healthy controls were stratified by the presence or absence of human leukocyte antigen DR β1 (*HLA-DRB1*) shared epitope. The numbers of the missing data were 6 (RA) and 15 (controls).

**Table 1:** Characteristics of the patients and healthy controls studied

	SLE	RA	Healthy controls
n	364	553	513
Male/female	21/343	43/510	238/275
Age*	42.8 ± 13.9	58.0 ± 11.3	34.1 ± 9.9

*Mean ± SD.

The control group consisted of healthy volunteers without any signs or symptoms of autoimmune diseases recruited at the same institutes.

This study was carried out in compliance with the Helsinki Declaration. The study was reviewed and approved by the research ethics committees of the University of Tsukuba, Sagamihara National Hospital and Juntendo University. Informed consent was obtained from all study participants.

### Genotyping

Genotyping of *TNIP1 *rs7708392 was carried out using the TaqMan genotyping assay (Applied Biosystems, Foster City, CA, USA), according to the manufacturer's instructions, using a TaqMan probe C__29349759_10*. HLA-DRB1 *was genotyped at the sequence level using a polymerase chain reaction (PCR) microtiter plate hybridization assay as previously described [20].

### Statistical analysis

A case control association study was conducted by χ^2 ^test using 2 × 2 contingency tables. The null hypotheses tested in this study were that there was no difference in the genotype or allele frequencies between all SLE patients and healthy controls, between SLE subsets and healthy controls, between all RA patients and all healthy controls, or between RA patients and healthy controls stratified by the presence or absence of *HLA-DRB1 *shared epitope.

The power to detect association was calculated on the basis of the frequency of the rs7708392C allele in Japanese healthy controls (69.9%). The sample size of this study (364 SLE patients, 553 RA patients and 513 controls) had the power of 80% to detect association when the genotype relative risk was1.36 (SLE) and 1.32 (RA) or greater, respectively [21].

To adjust for the gender difference between patients and healthy controls (Table 1), multiple logistic regression analyses were employed. The following were used as independent variables: for the genotypes of rs7708392, C/C = 1, C/G = 0, G/G = 0 under the recessive model for the C allele, C/C = 2, C/G = 1, G/G = 0 under the codominant model, and for gender, male = 0, female = 1.

The interaction between *TNIP1 *rs7708392 and *TNFAIP3 *rs2230926, which we recently replicated to be associated with SLE [10], was examined in 308 SLE, 372 RA and 449 healthy controls, using logistic regression analysis. Codominant (risk allele homozygotes *x_i _*= 2, heterozygotes *x_i _*= 1, nonrisk allele homozygotes *x_i _*= 0), dominant (risk allele homozygotes *x_i _*= 1, heterozygotes *x_i _*= 1, nonrisk allele homozygotes *x_i _*= 0) and recessive (risk allele homozygotes *x_i _*= 1, heterozygotes *x_i _*= 0, nonrisk allele homozygotes *x_i _*= 0) models for gene *i *were tested. The logistic regression model for interaction between gene *i *and gene *j *was given by logit(*P*) = *β*_0 _+ *β_i_x_i _+ β_j_x_j _*+ *β_ij_x_i_x_j_*. The deviation from 0 was evaluated for *b_ij _*by the Wald test. Population attributable risk percentage (PAR%) was estimated by the formula:

PAR%=Pe (RR−1)/(Pe [RR−1]+1)×100,

where Pe represents the risk genotype frequency in the population and RR represents the relative risk of the risk genotype [22]. Although RR cannot be determined from the case-control study design, it can be approximated by odds ratio (OR) when the incidence of the disease is sufficiently low. Because the incidence of SLE has been reported to be 3.0 in Japan and 1.8-7.6 in the United States per 100,000 persons per year [23] and is sufficiently small, OR can be adequately used for an approximation for RR. The PAR% in the Caucasian populations were calculated using the raw genotype count data for the previously reported study (cases: C/C 293, C/G 1,389, G/G 1,632, controls: C/C 735, C/G 4,510, G/G 7,050) [12].

## Results

### Replication of *TNIP1 *association with SLE in Japanese

The association of *TNIP1 *rs7708392 with SLE, recently demonstrated in the Caucasian (European-American and Swedish) populations [12], was examined in a Japanese population. Departure from Hardy-Weinberg equilibrium was observed neither in the cases nor in the controls (*P *> 0.3). As shown in Table 2, rs7708392C allele was significantly increased in Japanese SLE patients (76.5%) compared with healthy controls (69.9%, *P *= 0.0022, OR 1.40, 95% confidence interval [95% CI] 1.13-1.74), confirming the association in the Caucasians. The association was also detected under the recessive model for the rs7708392C allele (*P *= 0.0023, OR 1.52, 95% CI 1.16-2.00). Notably, the risk allele frequency was considerably greater in the Japanese (69.9%) than in the Caucasian healthy controls (24.3%) [12]. In the Japanese, PAR% was estimated to be 20.4% under the recessive model for the C allele (OR 1.52, population frequency of C/C 48.9%) and 31.0% under the dominant model (OR 1.50, population frequency of C/C + C/G 90.8%). These estimates were substantially greater than in the Caucasian populations, where the PAR% was 3.0% under the recessive model (OR 1.53, population frequency of C/C 6.0%) and 14.1% under the dominant model (OR 1.39, population frequency of C/C + C/G 42.7%).

**Table 2:** Association study of *TNIP1 *rs7708392 with SLE in a Japanese population

	Genotype			Allele	Allelic association		Recessive model*	
				
	C/C	C/G	G/G	C	*P*	OR (95%CI)	*P*	OR (95%CI)
All SLE (*n *= 364)	216 (59.3)	125 (34.3)	23 (6.3)	557 (76.5)	0.0022	1.40 (1.13-1.74)	0.0023	1.52 (1.16-2.00)
SLE subsets								
Renal disorder + (*n *= 203)	126 (62.1)	68 (33.5)	9 (4.4)	320 (78.8)	0.00065	1.60 (1.22-2.10)	0.0015	1.71 (1.23-2.38)
Neurologic disorder + (*n *= 53)	28 (52.8)	21 (39.6)	4 (7.5)	77 (72.6)	0.55	1.14 (0.73-1.79)	0.59	1.17 (0.66-2.05)
Serositis + (*n *= 55)	33 (60.0)	18 (32.7)	4 (7.3)	84 (76.4)	0.16	1.39 (0.88-2.20)	0.12	1.57 (0.89-2.75)
Anti-dsDNA Ab + (*n *= 280)	169 (60.4)	93 (33.2)	18 (6.4)	431 (77.0)	0.0026	1.44 (1.14-1.83)	0.0021	1.59 (1.18-2.13)
Anti-Sm Ab + (*n *= 67)	37 (55.2)	26 (38.8)	4 (6.0)	100 (74.6)	0.26	1.27 (0.84-1.91)	0.33	1.29 (0.77-2.15)
Onset <20 yr (*n *= 86)	46 (53.5)	34 (39.5)	6 (7.0)	126 (73.3)	0.37	1.18 (0.82-1.70)	0.43	1.20 (0.76-1.90)
Healthy controls (*n *= 513)	251 (48.9)	215 (41.9)	47 (9.2)	717 (69.9)		reference		reference

OR: odds ratio, 95% CI: confidence interval. Genotype and allele frequencies are shown in parentheses (%).

Association was tested by χ^2 ^analysis using 2 × 2 contingency tables. All SLE group as well as each SLE subset was compared with healthy controls.

*Association was tested under the recessive model for rs7708392C allele.

Because the female-to-male ratio was different between SLE patients and healthy controls (Table 1), we carried out multiple logistic regression analysis to examine the association after adjustment for gender. The association with SLE remained significant both under the recessive model for rs7708392C (*P *= 0.030, OR 1.40, 95% CI 1.03-1.89) and under the codominant model (*P *= 0.033, OR 1.30, 95% CI 1.02-1.65).

### Association of *TNIP1 *with Clinical Subsets of SLE

We next analyzed whether *TNIP1 *was associated with clinical subsets such as presence or absence of renal disorder, neurological disease, serositis, anti-dsDNA antibody, anti-Sm antibody, as well as the age of onset (< 20 yr). When the association was tested between patients having each phenotype and healthy controls, a tendency of stronger association was observed in the subsets with renal disorder and anti-dsDNA antibody as compared with all SLE (Table 2). These associations remained significant after adjustment for gender using logistic regression analysis (nephropathy positive versus controls: *P *= 0.0070, OR 1.50, 95% CI 1.12-2.01 under the codominant model and *P *= 0.011, OR 1.59, 95% CI 1.11-2.26 under the recessive model; anti-dsDNA positive versus controls: *P *= 0.033, OR 1.32, 95% CI 1.02-1.71 under the codominant model and *P *= 0.024, OR 1.45, 95% CI 1.05-2.00 under the recessive model).

On the other hand, significant association was not observed in the patient subsets having neurologic disease, serositis, anti-Sm antibody, and the patients with the age of onset <20 yr.

### Lack of Association with RA

We next tested association of *TNIP1 *rs7708392 with RA. Although a slight tendency toward association was observed, significant association with RA was not detected (Table 3). Significant association was not detected after the adjustment for gender (*P *= 0.847, OR 1.02, 95% CI 0.83-1.26 under the codominant model, *P *= 0.753, OR 1.04, 95% CI 0.80-1.36 under the recessive model), nor after stratification according to the presence or absence of *HLA-DRB1 *shared epitope (Table 3).

**Table 3:** Association study of *TNIP1 *rs7708392 with RA in a Japanese population

	Genotype			Allele	Allelic Association		Recessive Model*	
				
	C/C	C/G	G/G	C	*P*	OR (95%CI)	*P*	OR (95%CI)
All RA (*n *= 553)	292 (52.8)	215 (38.9)	46 (8.3)	799 (72.2)	0.23	1.12 (0.93-1.35)	0.21	1.17 (0.92-1.49)
All healthy controls (*n *= 513)	251 (48.9)	215 (41.9)	47 (9.2)	717 (69.9)		reference		reference
								
*HLA-DRB1 *shared epitope positive								
RA (*n *= 376)	203 (54.0)	142 (38.7)	31 (8.2)	548 (72.9)	0.76	1.04 (0.80-1.37)	0.57	1.11 (0.78-1.56)
Healthy controls (*n *= 202)	104 (51.5)	83 (41.1)	15 (7.4)	291 (72.0)		reference		reference
								
*HLA-DRB1 *shared epitope negative								
RA (*n *= 171)	86 (50.3)	70 (40.9)	15 (8.8)	242 (70.8)	0.67	1.07 (0.80-1.43)	0.68	1.08 (0.74-1.58)
Healthy controls (*n *= 296)	143 (48.3)	125 (42.2)	28 (9.5)	411 (69.4)		reference		reference

OR: odds ratio, 95% CI: confidence interval. Genotype and allele frequencies are shown in parentheses (%).

Association was tested by χ^2 ^analysis using 2 × 2 contingency tables. Comparisons were made between all RA and all healthy controls, HLA-DRB1 shared epitope positive RA and controls, and shared epitope negative RA and controls.

*Association was tested under the recessive model for rs7708392C allele.

### Lack of Evidence for Genetic Interaction between *TNFAIP3 *and *TNIP1*

Finally, we examined whether genetic interaction exists between *TNFAIP3 *and *TNIP1 *SNPs, because molecular interaction is known between the protein products of these genes. Although all combinations of the codominant, dominant and recessive models for each gene were examined, statistically significant gene-gene interaction was not detected (*P >*0.05).

## Discussion

In the present study, we replicated the association of *TNIP1 *rs7708392 with SLE in a Japanese population, which indicated that *TNIP1*, as well as *TNFAIP3*, is a susceptibility gene to SLE shared by the Caucasian and Asian populations. Because both TNIP1 and A20 are thought to be involved in the inhibition of NF-κB activation, genetic association of these genes implicates a causal role of NF-κB regulation pathway in the pathogenesis of SLE.

Kalergis et al. [24] demonstrated that expression of IκB-α, an inhibitor of NF-κB, was decreased in Fcγ receptor IIb-deficient mice which present lupus-like symptoms, and the symptoms were reduced by treatment with NF-κB inhibitors. Previous studies demonstrated that *TNFAIP3 *risk allele rs2230926G (127Cys) leads to reduced inhibitory activity of NF-κB activation [6] or reduced mRNA level of *TNFAIP3 *[10]. In view of these observations, it is speculated that the risk allele of *TNIP1 *may also be associated with reduced inhibitory activity of *TNFAIP3-TNIP1 *pathway.

*TNIP1 *rs7708392 is located in intron 1. Expression analysis using the GENEVAR [25] and the International HapMap databases [26] as previously described [27] did not show significant effect of rs7708392 genotypes on the mRNA level of *TNIP1 *(data not shown). Although the direct molecular mechanism of the risk allele to cause SLE remains unclear, it is possible that the risk allele may be associated with the selection of splicing variant. To date, at least 11 splice variants of *TNIP1 *have been identified [1]. Presence of alternative exon 1A and 1B, as well as splice variants lacking exon 2, has been described. Because rs7708392 is located between exon 1B and exon 2, it is possible that this SNP may influence the usage of the splicing isoform. It is also possible that other causative SNPs in tight LD with rs7708392 may exist. Such a possibility would be addressed by resequencing the entire *TNIP1 *gene.

Interestingly, in sharp contrast to the Caucasian populations, the risk rs7708392C constituted the major allele in the Japanese population. This resulted in substantially higher PAR% in the latter. We previously reported similar findings in *STAT4 *and *BLK *SNPs [14,15]. In Chinese, a SNP rs10036748, which is in tight LD with rs7708392 in Japanese (*r^2 ^*= 0.81, HapMap database [26]), has been shown to be associated with SLE. The frequencies of rs10036748 risk allele in Chinese (cases 79.7%, controls 66.1%) [8] are similar to those of rs7708392 in Japanese (Table 2). It should be noted that, because the information used to estimate the PAR% was based on the data from a variant that has not been shown to be the causal variant in *TNIP1*, and the estimates of the allele frequency and OR (as an approximation for RR) were taken from a rather small case-control study, the PAR% values shown here represent rough estimates. Nevertheless, the data suggest that the significance of *TNIP1 *in the genetic background of SLE may be substantially greater in the Asian than in the Caucasian populations.

In the association analysis with the clinical subsets, none of the case-only comparisons (cases with each clinical phenotype versus those without) reached statistical significance, partly because of the insufficient statistical power caused by the small sample size due to stratification. However, preferential association of *TNIP1 *with renal disorder and anti-dsDNA antibody was suggested by comparison with healthy controls. In our subjects, preferential association with renal disorder was also observed for *TNFAIP3 *[10].

On the other hand, association was not observed with the SLE subsets having neurological disease, serositis, anti-Sm antibody and age of onset <20. It is interesting to note that renal disorder and presence of anti-dsDNA are significantly correlated in SLE, while neurologic disorders are not, suggesting that these clinical features might represent different clinical subsets of SLE [28]. In view of this, our findings could be interpreted such that polymorphisms in *TNIP1*-*TNFAIP3 *pathway might play a significant role in the subset of SLE characterized by renal disorder and anti-dsDNA antibody, but not in the subset with neurologic disease. Such a hypothesis should be validated in future large-scale studies.

No strong evidence for association of rs7708392 with RA was obtained in this study. The sample size in this study (553 RA patients and 513 controls) provides 80% power to detect associations with genotype relative risk of 1.32 or greater, but we cannot rule out a possibility of weak association. Recently published meta-analysis of GWAS in Caucasians also failed to demonstrate statistically significant association of *TNIP1 *SNP with RA, although similarly to our observation, a tendency for association was detected [29]. Thus, while a role of *TNFAIP3 *is observed both in SLE and RA genetics, *TNIP1 *appears to play a major role in SLE, but not in RA. Such a difference might possibly imply that the molecular mechanism of *TNIP1 *association might not be fully explained by A20 modification. In support of this possibility, TNIP1 has been shown to block TNF-induced programmed cell death in *TNFAIP3 *deficient cells, indicating that TNIP1 does not always require A20 to perform its anti-apoptotic function [3]. Thus, further analysis on the molecular mechanisms involving these molecules is required.

## Conclusions

Association of *TNIP1 *with SLE was confirmed in a Japanese population. *TNIP1 *is a shared SLE susceptibility gene in the Caucasian and Asian populations, but the genetic contribution appeared to be greater in the Asians because of the higher risk allele frequency in the population. Taken together with the association of *TNFAIP3*, these observations underscore the crucial role of NF-κB regulation in the pathogenesis of SLE.

## Abbreviations

95%CI: 95% confidence interval; ABIN-1: A20-binding inhibitor of NF-κB -1; CI: confidence interval; GWAS: genome-wide association studies; HLA-DRB1: human leukocyte antigen DR β1; LD: linkage disequilibrium; NF-κB: nuclear factor-κB; OR: odds ratio; PAR%: population attributable risk percentage; PCR: polymerase chain reaction; RA: rheumatoid arthritis; RR: relative risk; SLE: systemic lupus erythematosus; SNP: single nucleotide polymorphism; TNFAIP3: tumor necrosis factor α-induced protein 3; TNIP1: TNFAIP3 interacting protein 1.

## Competing interests

RRG and TWB are employees of Genentech, Inc. (South San Francisco, CA, USA). The other authors declare that they have no competing interests.

## Authors' contributions

AK participated in the study design, carried out all genotyping and statistical analyses, and wrote the manuscript. JO carried out statistical analysis with AK and helped in the manuscript preparation. SI, HF, TH, DG, IM, MK, KM, ST, YT, HH and TS recruited Japanese patients with SLE and collected clinical information. RRG and TWB provided Caucasian data. NT designed and coordinated the study and helped in the manuscript preparation. All authors read and approved the final manuscript.

## References

[B1] Verstrepen L Carpentier I Verhelst K Beyaert R ABINs: A20 binding inhibitors of NF-κB and apoptosis signaling Biochem Pharmacol 2009 78 105 114 10.1016/j.bcp.2009.02.009 19464428

[B2] Fukushi M Dixon J Kimura T Tsurutani N Dixon MJ Yamamoto N Identification and cloning of a novel cellular protein Naf1, Nef-associated factor 1, that increases cell surface CD4 expression FEBS Lett 1999 442 83 88 10.1016/S0014-5793(98)01631-7 9923610

[B3] Oshima S Turer EE Callahan JA Chai S Advincula R Barrera J Shifrin N Lee B Benedict Yen TS Woo T Malynn BA Ma A ABIN-1 is a ubiquitin sensor that restricts cell death and sustains embryonic development Nature 2009 457 906 909 10.1038/nature07575 19060883 PMC2642523

[B4] Thomson W Barton A Ke X Eyre S Hinks A Bowes J Donn R Symmons D Hider S Bruce IN Wellcome Trust Case Control Consortium Wilson AG Marinou I Morgan A Emery P YEAR Consortium Carter A Steer S Hocking L Reid DM Wordsworth P Harrison P Strachan D Worthington J Rheumatoid arthritis association at 6q23 Nat Genet 2007 39 1431 1433 10.1038/ng.2007.32 17982455 PMC2674282

[B5] Plenge RM Cotsapas C Davies L Price AL de Bakker PI Maller J Pe'er I Burtt NP Blumenstiel B DeFelice M Parkin M Barry R Winslow W Healy C Graham RR Neale BM Izmailova E Roubenoff R Parker AN Glass R Karlson EW Maher N Hafler DA Lee DM Seldin MF Remmers EF Lee AT Padyukov L Alfredsson L Coblyn J Two independent alleles at 6q23 associated with risk of rheumatoid arthritis Nat Genet 2007 39 1477 1482 10.1038/ng.2007.27 17982456 PMC2652744

[B6] Musone SL Taylor KE Lu TT Nititham J Ferreira RC Ortmann W Shifrin N Petri MA Kamboh MI Manzi S Seldin MF Gregersen PK Behrens TW Ma A Kwok PY Criswell LA Multiple polymorphisms in the *TNFAIP3 *region are independently associated with systemic lupus erythematosus Nat Genet 2008 40 1062 1064 10.1038/ng.202 19165919 PMC3897246

[B7] Graham RR Cotsapas C Davies L Hackett R Lessard CJ Leon JM Burtt NP Guiducci C Parkin M Gates C Plenge RM Behrens TW Wither JE Rioux JD Fortin PR Graham DC Wong AK Vyse TJ Daly MJ Altshuler D Moser KL Gaffney PM Genetic variants near *TNFAIP3 *on 6q23 are associated with systemic lupus erythematosus Nat Genet 2008 40 1059 1061 10.1038/ng.200 19165918 PMC2772171

[B8] Han JW Zheng HF Cui Y Sun LD Ye DQ Hu Z Xu JH Cai ZM Huang W Zhao GP Xie HF Fang H Lu QJ Xu JH Li XP Pan YF Deng DQ Zeng FQ Ye ZZ Zhang XY Wang QW Hao F Ma L Zuo XB Zhou FS Du WH Cheng YL Yang JQ Shen SK Li J Genome-wide association study in a Chinese Han population identifies nine new susceptibility loci for systemic lupus erythematosus Nat Genet 2009 41 1234 1237 10.1038/ng.472 19838193

[B9] Shimane K Kochi Y Horita T Ikari K Amano H Hirakata M Okamoto A Yamada R Myouzen K Suzuki A Kubo M Atsumi T Koike T Takasaki Y Momohara S Yamanaka H Nakamura Y Yamamoto K The association of a nonsynonymous single-nucleotide polymorphism in *TNFAIP3 *with systemic lupus erythematosus and rheumatoid arthritis in the Japanese population Arthritis Rheum 2010 62 574 579 10.1002/acr.20194 20112363

[B10] Kawasaki A Ito I Ito S Hayashi T Goto D Matsumoto I Takasaki Y Hashimoto H Sumida T Tsuchiya N Association of *TNFAIP3 *polymorphism with susceptibility to systemic lupus erythematosus in a Japanese population J Biomed Biotechnol 2010 2010 207578 20617138 10.1155/2010/207578 PMC2896654

[B11] Nair RP Duffin KC Helms C Ding J Stuart PE Goldgar D Gudjonsson JE Li Y Tejasvi T Feng BJ Ruether A Schreiber S Weichenthal M Gladman D Rahman P Schrodi SJ Prahalad S Guthery SL Fischer J Liao W Kwok PY Menter A Lathrop GM Wise CA Begovich AB Voorhees JJ Elder JT Krueger GG Bowcock AM Abecasis GR Genome-wide scan reveals association of psoriasis with IL-23 and NF-κB pathways Nat Genet 2009 41 199 204 10.1038/ng.311 19169254 PMC2745122

[B12] Gateva V Sandling JK Hom G Taylor KE Chung SA Sun X Ortmann W Kosoy R Ferreira RC Nordmark G Gunnarsson I Svenungsson E Padyukov L Sturfelt G Jönsen A Bengtsson AA Rantapää-Dahlqvist S Baechler EC Brown EE Alarcón GS Edberg JC Ramsey-Goldman R McGwin G Jr Reveille JD Vilá LM Kimberly RP Manzi S Petri MA Lee A Gregersen PK A large-scale replication study identifies *TNIP1*, *PRDM1*, *JAZF1*, *UHRF1BP1 *and *IL10 *as risk loci for systemic lupus erythematosus Nat Genet 2009 41 1228 1233 10.1038/ng.468 19838195 PMC2925843

[B13] Remmers EF Plenge RM Lee AT Graham RR Hom G Behrens TW de Bakker PI Le JM Lee HS Batliwalla F Li W Masters SL Booty MG Carulli JP Padyukov L Alfredsson L Klareskog L Chen WV Amos CI Criswell LA Seldin MF Kastner DL Gregersen PK *STAT4 *and the risk of rheumatoid arthritis and systemic lupus erythematosus N Engl J Med 2007 357 977 986 10.1056/NEJMoa073003 17804842 PMC2630215

[B14] Kawasaki A Ito I Hikami K Ohashi J Hayashi T Goto D Matsumoto I Ito S Tsutsumi A Koga M Arinami T Graham RR Hom G Takasaki Y Hashimoto H Behrens TW Sumida T Tsuchiya N Role of STAT4 polymorphisms in systemic lupus erythematosus in a Japanese population: a case-control association study of the *STAT1-STAT4 *region Arthritis Res Ther 2008 10 R113 10.1186/ar2516 18803832 PMC2592800

[B15] Ito I Kawasaki A Ito S Hayashi T Goto D Matsumoto I Tsutsumi A Hom G Graham RR Takasaki Y Hashimoto H Ohashi J Behrens TW Sumida T Tsuchiya N Replication of the association between *C8orf13-BLK *region and systemic lupus erythematosus in a Japanese population Arthritis Rheum 2009 60 553 558 10.1002/art.24246 19180478

[B16] Ito I Kawasaki A Ito S Kondo Y Sugihara M Horikoshi M Hayashi T Goto D Matsumoto I Tsutsumi A Takasaki Y Hashimoto H Matsuta K Sumida T Tsuchiya N Replication of association between *FAM167A(C8orf13)-BLK *region and rheumatoid arthritis in a Japanese population Ann Rheum Dis 2010 69 936 937 10.1136/ard.2009.118760 19740902

[B17] Gallagher J Howlin J McCarthy C Murphy EP Bresnihan B FitzGerald O Godson C Brady HR Martin F Identification of Naf1/ABIN-1 among TNF-α-induced expressed genes in human synoviocytes using oligonucleotide microarrays FEBS Lett 2003 551 8 12 10.1016/S0014-5793(03)00823-8 12965196

[B18] Hochberg MC Updating the American College of Rheumatology revised criteria for the classification of systemic lupus erythematosus Arthritis Rheum 1997 40 1725 10.1002/art.1780400928 9324032

[B19] Arnett FC Edworthy SM Bloch DA McShane DJ Fries JF Cooper NS Healey LA Kaplan SR Liang MH Luthra HS Medsger TA Jr Mitchell DM Neustadt DH Pinals RS Schaller JG Sharp JT Wilder RL Hunder GG The American Rheumatism Association 1987 revised criteria for the classification of rheumatoid arthritis Arthritis Rheum 1988 31 315 324 10.1002/art.1780310302 3358796

[B20] Kawai S Maekawajiri S Tokunaga K Kashiwase K Miyamoto M Akaza T Juji T Yamane A Routine low and high resolution typing of the HLA-DRB gene using the PCR-MPH (microtitre plate hybridization) method Eur J Immunogenet 1996 23 471 486 10.1111/j.1744-313X.1996.tb00137.x 8971544

[B21] Ohashi J Yamamoto S Tsuchiya N Hatta Y Komata T Matsushita M Tokunaga K Comparison of statistical power between 2 × 2 allele frequency and allele positivity tables in case-control studies of complex disease genes Ann Hum Genet 2001 65 197 206 10.1017/S000348000100851X 11434330

[B22] Cole P MacMahon B Attributable risk percent in case-control studies Br J Prev Soc Med 1971 25 242 244 5160433 10.1136/jech.25.4.242 PMC478665

[B23] Rus V Hajeer A Hochberg MC Silman AJ, Hochberg MC Systemic lupus erythematosus Epidemiology of the Rheumatic Diseases 2001 2 Oxford: Oxford University Press 123 140

[B24] Kalergis AM Iruretagoyena MI Barrientos MJ González PA Herrada AA Leiva ED Gutiérrez MA Riedel CA Bueno SM Jacobelli SH Modulation of nuclear factor-κB activity can influence the susceptibility to systemic lupus erythematosus Immunology 2009 128 e306 e314 10.1111/j.1365-2567.2008.02964.x 19016912 PMC2753943

[B25] GENEVAR - GENe Expression VARiation http://www.sanger.ac.uk/humgen/genevar/

[B26] International HapMap Project http://hapmap.ncbi.nlm.nih.gov/index.html.en

[B27] Kawasaki A Kyogoku C Ohashi J Miyashita R Hikami K Kusaoi M Tokunaga K Takasaki Y Hashimoto H Behrens TW Tsuchiya N Association of *IRF5 *polymorphisms with systemic lupus erythematosus in a Japanese population. Support for a crucial role of intron 1 polymorphisms Arthritis Rheum 2008 58 826 834 10.1002/art.23216 18311811

[B28] Taylor KE Remmers EF Lee AT Ortmann WA Plenge RM Tian C Chung SA Nititham J Hom G Kao AH Demirci FY Kamboh MI Petri M Manzi S Kastner DL Seldin MF Gregersen PK Behrens TW Criswell LA Specificity of the *STAT4 *genetic association for severe disease manifestations of systemic lupus erythematosus PLoS Genet 2008 4 e1000084 10.1371/journal.pgen.1000084 18516230 PMC2377340

[B29] Stahl EA Raychaudhuri S Remmers EF Xie G Eyre S Thomson BP Li Y Kurreeman FAS Zhernakova A Hinks A Guiducci C Chen R Alfredsson L Amos CI Ardlie KG BIRAC Consortium Barton A Bowes J Brouwer E Burtt NP Catanese JJ Coblyn J Coenen MJH Costenbader KH Criswell LA Crusius JBA Cui J de Bakker PIW De Jager PL Ding B Genome-wide association study meta-analysis identifies seven new rheumatoid arthritis risk loci Nat Genet 2010 42 508 514 10.1038/ng.582 20453842 PMC4243840

